# What Do Consumers Need Before, During and After a Patient Safety Incident Review? A Qualitative Study

**DOI:** 10.1111/hex.70646

**Published:** 2026-03-26

**Authors:** Mia Bierbaum, Yinghua Yu, Charlotte J. Molloy, Lorelle Bowditch, Peter D. Hibbert

**Affiliations:** ^1^ Australian Institute of Health Innovation, Faculty of Medicine, Health and Human Sciences Macquarie University Sydney New South Wales Australia; ^2^ IIMPACT in Health, Allied Health and Human Performance, College of Health Adelaide University Adelaide South Australia Australia

**Keywords:** adverse events, consumer voice, hospital incident reporting, hospitals, incident review, medical errors, patients, patient participation, patient safety

## Abstract

**Objectives:**

When a patient (consumer) suffers serious harm that is considered preventable, healthcare organisations typically conduct a review (or investigation) of the incident to better understand what happened and why, and take action to prevent similar incidents from reoccurring. These reviews and associated other organisational responses can compound psychological harm to consumers. Understanding how consumer needs can be met during these processes may assist in reducing this harm. This study aimed to understand what is important to consumers before, during and after patient safety incident reviews, as framed by their experiences.

**Methods:**

Participants were purposely recruited through patient advocacy organisations, including newsletter advertisements, and through consumer representatives. Semi‐structured interviews were conducted over video conference with consumers and consumer representatives between April 2024 and March 2025, and transcribed verbatim. The data analysis utilised grounded theory techniques, including inductive analysis, theoretical saturation and constant comparison, to identify key needs and perspectives of consumers during patient safety incident reviews.

**Results:**

A total of 28 participants were recruited and participated in 21 interviews and one focus group, across four states and territories in Australia (New South Wales, Victoria, the Australian Capital Territory and Queensland). Analysis identified that consumers want more empathetic, practical and inclusive support throughout patient safety incident reviews, with clearer communication, transparency and accountability. Consumers want their voices heard, valued and documented across the review. They also want opportunities for greater and more flexible involvement in the review process, and a structured mechanism to track, report and communicate implementation changes, driven by a desire to prevent patient safety incidents from recurring.

**Conclusions:**

Ensuring the response to incidents is more patient‐centred, through more meaningful engagement and consideration of consumers' emotional, psychological, communication and transparency, accountability and practical needs, is an important way forward. A patient‐centred relational approach, complementing systems‐based reviews, will contribute to learning and improvement following an incident, enhance consumers' experience, including reducing compounded harm, and support them in regaining trust in the healthcare system.

**Patient or Public Contribution:**

There were 28 consumers with experience of patient safety reviews or as consumer representatives interviewed. This study is part of a 4‐year project that includes a research advisory committee comprised of six consumers from four jurisdictions. They are involved throughout the project lifecycle and advise the project team on methodology and the broad interpretation of the data across the project. In this study, they were invited to be study participants.

## 1 Introduction

Reviews of patient safety incidents (PSI) (harm that occurs during hospital care) are a common method used internationally to prevent future incidents from occurring [1]. Their purpose is to understand what happened during the PSI and how, to identify factors to be addressed to reduce the risk of reoccurrence and learn from the PSI [2].

Engagement with patients and families (and carers), during PSI reviews (also known as investigations), is recommended across many jurisdictions in Australia [3, 4, 5] and internationally [6]. In an Australian healthcare context, the term ‘patient’ refers to a person receiving care, and ‘consumer’ refers to a person who has used (or may use) health services, including their family or carer [7]. In this paper, we use ‘consumers’ as an umbrella term to capture the perspectives of patients, and/or their families and carers. Key benefits of consumer input into the review process include incorporation of a unique perspective of the PSI [8], reestablishing a relationship and regaining trust in the healthcare provider [9], as well as enhanced consumer satisfaction, engagement and ownership [3]. The PSI review process has the potential to compound psychological harm for consumers [2], so increasing consumer engagement to better understand their needs is an important step toward reducing this harm.

In some jurisdictions, consumers are supported by a Dedicated Family Contact (DFC) (New South Wales) [10] or a consumer liaison officer through the duty of candour process (Victoria) [11]. Similar support is provided to consumers across Australian states and territories. DFCs and liaison officers are staff members assigned as points of contact during a PSI review to provide regular communication and support consumers in navigating the process [10, 11]. In some circumstances, consumers are actively engaged and encouraged to provide input on the review findings and recommendations, while in other circumstances, they are simply provided with a final report or summary outlining the review process and findings [2]. Consumer engagement in the review process is variable globally, with numerous barriers limiting opportunities for them to provide input into PSI review findings, recommendations and improvement plans [2, 8, 12]. Barriers to consumer involvement include trauma, limited personal support, ongoing medical needs, limited health literacy and understanding of patient safety, as well as limited time and availability. Limited support for consumer perspectives or engagement from healthcare professionals, as well as the cost and time involved in actively engaging consumers, potential legal implications, and broader organisational policies and culture, can limit engagement [12].

Parallel research with this current study has identified that healthcare staff in Australia perceive a need for increased engagement with consumers, with support to incorporate their input and perspectives into PSI reviews [13]. This includes the consideration of consumer needs when determining the type and depth of review methodology to be utilised [13]. In Australia, the depth of review and methodology used is typically governed by state‐ and territory‐specific policies and legislation, guided by a standardised measure of harm experienced by the patient [3, 14]. There appears to be a wide variation as to whether consumer input is sought or considered during this process [13], including whether consumer representatives (CRs) (people who provide a consumer perspective and advocate for consumer interests [7]) are involved in the review process [15].

In order to support engagement of consumers during PSI reviews in Australia, a greater understanding of their needs throughout the process is required. This qualitative study aims to understand what consumers need before, during and after PSI reviews in the Australian healthcare context, informed by their perspectives and experiences of the process. These findings, in conjunction with findings from parallel studies being conducted within our broader research programme [13, 15, 16, 17, 18], will inform the development of best practice principles that will, in turn, guide policy recommendations for patient safety reviews to adopt a more consumer‐oriented approach. These principles will also guide a codesign and feasibility study, which will trial a re‐engineered process for patient safety reviews.

## 2 Methods

### 2.1 Study Type

This qualitative study comprises one arm of a National Health and Medical Research Council (NHMRC) Partnership Grant project [16] broadly aiming to investigate ways to improve the health systems' responses when patients are harmed. This study aimed to elucidate consumer needs and experiences associated with a PSI review. Qualitative data were collected through a focus group and interviews with participants across four state and territory health systems: New South Wales (NSW), Victoria (VIC), the Australian Capital Territory (ACT), and Queensland (QLD). The reporting of this study was guided by the Consolidated Criteria for Reporting Qualitative Research (COREQ) framework [19] (Supporting Information S1).

### 2.2 Ethics Approval

This study received human research ethics approval from the Northern Sydney Local Health District (NSLHD) Human Research Ethics Committee, approval number 2023/ETH02341.

### 2.3 Recruitment and Setting

For the purposes of describing participants, consumers were those who had experienced a PSI themselves (as a patient) or were family members of a patient who had. Participants were considered CRs if they had been involved in a PSI review panel or had represented consumers on patient safety committees or within advocacy or health organisations, with or without a personal experience of a PSI. Participants were recruited using a non‐probability sampling strategy, combining purposive and convenience sampling with self‐selection. Recruitment occurred across four Australian jurisdictions (NSW, VIC, ACT and QLD). Jurisdictional health service staff invited eligible participants via phone, and followed up with a formal email invitation, including the study participant information, consent form and study team details. Additional consumers were invited to participate via a study advertisement in a consumer organisation newsletter. These consumers were asked to contact the study team to register their interest. To support participation of consumers from different backgrounds, we offered assistance for consent processes via phone or email. We also offered assistance when requested for the interviews, including Auslan (Australian Sign Language) interpreters. The total number of people invited to participate is unknown. All participants provided informed and written consent.

Participants were compensated with an honorarium of AUD 40. This was intended to reimburse them for their time, not as an incentive to participate, in line with best ethical practices.

### 2.4 Data Collection

Semi‐structured interviews were conducted via videoconference or phone, audio‐recorded and transcribed verbatim. One focus group was also conducted (via videoconference) for convenience with a group already known to one another. This focus group followed the same discussion guide and analysis as the interviews and was considered together in the analysis. The focus group and interviews were conducted by researchers P. D. H. (professor; male), M. B. (PhD, Research fellow; female), and Y. Y. (PhD, Research fellow; female), who are experienced interviewers and health service qualitative researchers. Some interviewees were known to the lead researcher (P. D. H.) through prior professional networks; however, participation was entirely voluntary, and individuals were under no obligation to take part in the study. Neither M. B. nor Y. Y. had a prior relationship with the interviewees before data collection. The interview topic guide included questions about the criteria used to determine how PSIs are reviewed, consumer needs before, during and after a PSI review, including the open disclosure process, and the formulation of recommendations and implementation of change. The interview schedule was informed by the literature and is shown in Supporting Information S2. Participants completed a short survey providing demographic details (location, age and gender).

### 2.5 Data Analysis

This study employed grounded theory techniques, including inductive analysis [20], theoretical saturation [21] and constant comparison [22]. Theoretical and analytic notes were taken throughout the interviews and focus group by two experienced qualitative researchers (M. B. and Y. Y.), who independently recorded reflexive and theoretical notes during the fieldwork. Individually, they captured emerging concepts, patterns and points of analytic interest. These notes informed the development of preliminary themes. M. B. is an implementation scientist and experienced qualitative researcher. Their academic research has focused on health professionals and consumer perspectives across a broad spectrum of health conditions. Y. Y. is a sociologist with expertise in qualitative research. Their disciplinary backgrounds informed and shaped the analysis to focus on how consumer needs are articulated, interpreted, and potentially constrained within institutional, organisational, and policy contexts. P. D. H. has 20 years of experience in the field of patient safety reviews. P. D. H. did not participate in the analysis as they felt that they may bias the analysis with some of their preconceptions – instead, they provided sense‐checking to the analysis. Inductive thematic analysis [23] of the transcripts was conducted in NVivo 14 software [24] following each interview. Each transcript was read for familiarity and coded to identify broad themes by two coders (M. B. and Y. Y.). The final themes were discussed, and a thematic framework was outlined through team consensus with iterative engagement with both the interview data and analytic notes. Transcripts were then recoded to ensure consistency with the thematic framework.

The study reached theoretical data saturation; however, more interviews were conducted after this point. This is because the study is part of a large research grant across four Australian jurisdictions (NSW, VIC, QLD, ACT) [16] and the researchers were keen to be inclusive across jurisdictions. The research was conducted sequentially across the jurisdictions, partially by design and partially by timing of the relevant approval committees. Therefore, even though we seemed to reach theoretical saturation after interviews/focus groups with two jurisdictions, we continued interviewing to ensure representation from all jurisdictions.

## 3 Results

### 3.1 Demographics

A total of 28 participants were recruited and participated in 22 interviews and 1 focus group. The focus group was attended by five participants, and all interviews were conducted individually, with the exception of one session, which involved two participants with a familial relationship. Participants included consumers who had experienced a PSI or who had supported a family member through a PSI (*n* = 12), or were CRs (*n* = 21), with overlap between each group. The majority of participants were based in VIC (*n* = 11, 39%) and QLD (*n* = 8, 29%), female (*n* = 17, 68%), and all were aged 40 or over (Table 1). Interviews lasted an average of 54 min, ranging from 31 to 69 min, and the focus group lasted 59 min totalling just under 21 h of data collection. Exemplar quotes labelled with participant codes are provided to support the thematic framework. To maintain anonymity, demographic data have not been presented alongside quotes.

**Table 1:** Demographic details of participating consumers and consumer representatives.

Characteristic	*n*	%
State		
VIC	11	39%
QLD	8	29%
ACT	6	21%
NSW	3	11%
Age*		
< 40 years	6	23%
40–49 years	5	19%
50–59 years	7	27%
> 60 years	8	31%
Gender*		
Female	17	65%
Male	8	31%
Non‐binary	1	4%

*Note:* The qualitative analysis identified consumer needs framed by their experiences before, during, and after the PSI review process. Some of the needs clearly cut across all of these stages.

* Note that two participants did not report their gender or age.

### 3.2 Consumer Needs Before the Review

Participants reported that consumers want to be taken seriously, genuinely listened to, and for health service staff to show empathy towards them, right from their initial engagement after the PSI. This helps to develop trust in the health organisation and the review process, and facilitates greater consumer engagement and satisfaction with the PSI review process.

It's more about just really understanding, getting the feeling that we're not being fobbed off [dismissed], we're being listened to, somebody's taking us seriously, and explaining to us what had happened there.

(Participant 12, consumer, interview)

Participants felt that organisations should also acknowledge the PSI and their intent to review why it occurred, as well as provide a clear and sincere apology. They felt that this process is important for regaining consumer trust in the organisation and for providing reassurance that action will be taken. The review process was considered key to help consumers find closure and experience a sense of justice.

The responses are “Oh, we know. We're deeply sorry” … They lack the sincerity, you know. I mean, that's just a superficial thing, but I think that the first piece of correspondence that you get that first interaction is absolutely critical. That is where the trust, I mean, really you've got to also acknowledge that you're starting from a point of zero trust … That initial engagement is absolutely critical to establish some form of commitment and to start strengthening trust, because the journey you go on requires enormous trust.

(Participant 11, consumer representative, interview)

Participants explained that consumers need practical, tangible support to prepare for the review process. Participants reported experiencing varied support during PSI reviews, with some being guided through the process smoothly and others being provided with no support or information. The provision of a DFC, patient advocate, or someone who can support consumers throughout the review process was considered important, particularly to explain what they should expect throughout the PSI review process. Participants highlighted that support should be provided by someone who understands consumer needs.

[What we really need is] a dedicated person or a, you know, a phone number, a team to contact so you leave the hospital, you're given a pamphlet with “Please, you know, in the next days, person X will call you to check in” and just, I don't know, just a team of people that you can e‐mail once a week or something and say “hey.”

(Participant 8, consumer, interview)

Participants noted that practical information, such as information about consumer rights, where they can access support, and who they need to contact to make and escalate complaints, is also needed.

I think the root of the issue is knowing what their consumer rights are in the first place. So, a lot of people are obviously hesitant to navigate different systems … navigating [the health system] is already difficult.

(Participant 10, consumer representative, focus group)

Consideration and consultation with the consumer to understand whether and to what degree they want to be involved in the review was deemed critical by participants. The timing of health services' attempts to communicate was also seen as important, with consumers often shrouded in a fog of illness, exhaustion or grief, and unable to fully engage immediately following the PSI.

In that meeting, which was two days after I made my complaint, [patient] was still sick. She was still very sick. I didn't think at that point that she was going to live, but she was still really sick. So, that was too early.

(Participant 2, consumer, interview)

Some participants reported feeling that health services assumed they would prefer not to engage in the review process or that they were too vulnerable to participate.

It's also very common in [state], all the health services, to put forward, literally in writing, their excuse for not involving consumers saying, “Oh, it would be upsetting for the consumer.”

(Participant 1, consumer representative, interview)

Participants felt that consumers' preferences and needs should be considered in relation to what they want the review to achieve, as well as their access to technology to communicate, and access to face‐to‐face meetings.

The people involved need to be asked, you know, do they want to have their meetings online? Do they want to have them in person? Do they want an e‐mail or a phone call? Do they need to use assistive technology to communicate? You know, just all of those things that are about, yeah, being able to communicate with people in the way that they can best do it.

(Participant 5, consumer representative, focus group)

Consideration of the specific needs of consumers, including culturally and linguistically diverse (CALD) people and those with a disability, was also deemed important by participants. This included acknowledgement of their cultural and social practices, language barriers, familiarity with the Australian health system, and tailored supports they may need, such as having consistent access to interpreters.

I seem to always ask … what about CALD communities and patients and carers? They [Healthcare organisations] sort of shut it down and say, “Yes, you know, we'll have an interpreter.” But … the quality of that interaction is what is missing…you certainly don't want … [to assume] … that you've given them the report and it's translated that they actually understand it because sometimes their written and their spoken literacy levels are very different.

(Participant 14, consumer representative, interview)

…[what is needed is] a support person or a support worker to help understand the what's happened with situations that have occurred. So, nurses, doctors, reception, all the hospital staff, quite often, don't understand deaf people or deaf culture. So yes, there is quite a bit of trauma in Deaf people, generally speaking, because you know that the survivors are really upset about the situations happening.

(Participant 20, consumer, interview)

Participants felt it was important to consider consumer concerns regarding whether a review ought to be conducted, and to what depth, when deciding which review methodology will be used to investigate a PSI, with several reporting that they felt all PSIs should be investigated comprehensively.

I would expect that there'd be a level of review at that point based on just my concerns, or my family's concerns. And that would be perhaps whether serious harm had happened or not. Maybe it would be a case of, like, if it was a near miss.

(Participant 3, consumer representative, interview)

Participants noted concern about the level of discretion healthcare organisations have about the depth of PSI reviews, typically about the use of rapid review for low‐harm PSIs and near misses. They felt that near misses were underreported and that they warranted more in‐depth review. Several participants felt that rapid reviews did not take into account the consumer perspective of the PSI adequately. Consumer perspectives on the level of harm were identified as an important consideration for all PSI reviews. Participants also noted that frequency and preventability of a PSI, or near miss, should be considered.

I think the severity of actual harm, is the wrong approach. I think I've always been concerned about the notion that sometimes the mechanism of the error and what's happened could have … caused really severe harm. But there was some kind of lucky break, you know, that turned it into a “near miss.” And yet there's clearly a high risk in the system there that needs to be dealt with and the fact that he kind of got lucky and didn't end up horribly wrong is really not a logical or rational reason not to investigate it.

(Participant 1, consumer representative, interview)

At times, participants felt they had to push health organisations to take their needs and perspectives seriously. This suggests the need for institutional care that recognises and addresses potential power imbalances.

Patients or family members feel like the patient has experienced harm and then it [the health service] doesn't take it seriously. … I think a lot of the time when there's a perception that there has been some kind of harm done, usually the patient or family member or carer have to really kind of push to get investigated. You have to speak up and advocate for yourself.

(Participant 17, consumer, interview)

In summary, before a review, consumers need to be shown empathy, to be taken seriously, and listened to, and to be provided with a clear and sincere apology to facilitate trust and reassurance in the process. Support before a review involves actively checking for willingness to participate, offering flexibility in terms of timing and formats of engagement early on. Consumers should be asked what they hope the review will achieve. Practical supports, such as a patient advocate or a DFC, can provide consistent, clear information about the review process, timelines and expectations. Support should be inclusive and appropriate for people with diverse needs, including those from CALD backgrounds and people with disabilities.

### 3.3 Consumer Needs During the Review

Participants highlighted that consumers are largely driven to be involved in the PSI review process by their desire to prevent PSIs from reoccurring with other patients. This implies the importance of keeping consumers informed to honour their motivations.

I want to know the outcome of this because this is unacceptable and cannot happen again.

(Participant 7, consumer, interview)

A lot of the reason they're going into it is because they just don't want this to happen again. And so, closing that feedback really involves telling them these are the ways that we are working to make sure it won't happen again.

(Participant 15, consumer representative, focus group)

Participants observed that consumers have specific support and communication needs that should be attended to throughout a PSI review. Transparency around the review process, progress, and findings was considered vital. This could help to set clear expectations for consumers about their psychological, emotional and financial (e.g., parking and transport) engagement.

…initially, they [the health service] said that they'll … [provide] … bimonthly sort of updates. I said no, I would rather have a fortnightly [update] even if there's no information. That's fine. At least you're touching base with us.

(Participant 9, consumer, interview)

It is also important to be mindful of the risk of compounded harm during the review process, and to clearly communicate timelines so that consumers understand what will happen and when. This suggests that transparency and accountability are not administrative details, but safeguards against further harm during the review.

…I would have liked to be able to sit down and do like a step‐by‐step kind of breakdown of how it all went so wrong … I don't understand how it can be a comprehensive clinical incident review if they don't even know at what time [the incident occurred].

(Participant 17, consumer, interview)

Some participants noted poor transparency and communication around the review process, the identification of findings, and recommendation formulation and implementation.

It's [the review] all written in a way where it's like the responsibility for the recommendations is diffused among a faceless crowd. You don't know the names, so it's all diffused and there's no one person to blame, which is good in a way, I know. No blame culture, but it's … not working like that because it's a way out of actual responsibility.

(Participant 2, consumer representative, interview)

Clear, detailed and timely communication was perceived to help consumers know what to expect during the review process and how they can contribute. It also helped increase consumers' understanding of projected timelines and review plans, and provided updates on review progress and the implementation of recommendations. Participants expressed a desire for regular communication and reassurance that they had not been forgotten by the health organisations involved.

Knowing the time frames of when to expect to hear back as well, it's really important.

(Participant 10, consumer representative, focus group)

We just want to know, are the wheels turning? We're not letting you sweep this under the rug. You know, I don't care if nothing happened this week, but you tell us, you know?

(Participant 8, consumer, interview)

Equally important is the timing of these communications. Communication should not disrupt ongoing patient care and should be sensitive to the emotional needs of consumers. When communicating with consumers, health services should be sensitive to the psychological and emotional stress that the consumer may be experiencing.

You're [the health service] in big trouble, but right now, can you just help my son? And can we then revisit this later? That is what I would have liked to have happened. Then I would have liked to have had an explanation as to what they then do after that, and not that this [the incident] would be thoroughly investigated and we will get back to you … at the time I was going through it, I'm like, “yeah, whatever, I just want my son well.”

(Participant 7, consumer, interview)

Support from a DFC or similar role, together with written explanations of the process and consumers' rights in brochures, was suggested as a useful tool to provide this information to consumers. Participants noted how important it is that consumers' health literacy levels are considered and accounted for when providing information and support. The format and content of the PSI review report and other communication with consumers must reflect these needs. Participants want PSI review reports to be written in a format that is both comprehensive and user‐friendly, and in an accessible format, which may include translations into different languages. Participants felt reports should also provide lay summaries and explanations of medical jargon throughout.

…the main thing which we need is a dedicated person who addresses anything. A time frame or a structure – that things will happen at a certain point and information's followed through.

(Participant 9, consumer, interview)

Some participants emphasised that people from marginalised groups and communities needed greater support. In their experiences, multicultural communities were reluctant to reach out, partly due to culturally related issues, mistrust or prior negative experiences.

I think it's probably fair to say we don't necessarily get multicultural community members reaching out to our organisation in the same frequency that … people who don't come from multicultural communities do … I think the root of the issue is knowing what their consumer rights are in the first place. A lot of people are obviously hesitant to different systems, so already navigating it is already difficult when something traumatic happens.

(Participant 10, consumer representative, focus group)

Language barriers and limited health literacy are concerns for CALD communities, as highlighted by one participant below. Those constraints, if not addressed appropriately, can lead to confusion, disengagement and a sense of exclusion from the review process:

I often wonder what happens with people that can't speak English. It can be an isolating experience, and you speak the language. If you don't speak the language, is it all too hard to get interpreters involved? Do the interpreters have the appropriate skill level to be dealing with … a traumatic outcome? Do they show the appropriate empathy as well?

(Participant 14, consumer representative, interview)

Consumers with a disability introduce additional layers of complexity, requiring appropriate and tailored support. One participant shared their experiences related to access to interpreters with appropriate skills and health literacy:

Quite often, they've [health services] got, you know, people who are interpreting who perhaps aren't appropriately skilled. So incorrect medical information [is provided] … we need appropriately qualified interpreters to be interpreting for us.

(Participant 20, consumer, interview)

Nearly all participants indicated that they could have been better supported throughout the various stages of the PSI review process. While participants had diverse experiences, a consistent theme was the importance of empathy and compassion throughout the entire review process, to support consumers who may feel vulnerable. Ideally, there should be counselling services available for those in need during the review process.

…not everyone will need that level of intervention, but they should be a way that they can refer people to some form of counselling to support them.

(Participant 6, consumer, focus group)

Participants reported that communication about reviews can be bureaucratic, defensive, subjective and lack empathy. Participants recounted receiving formulaic letters outlining basic summaries of the findings from a review, without any detail about the findings that were identified, or what would be changed to prevent the issue from reoccurring.

You get back that bureaucratic waffle that doesn't actually achieve anything, and that's how I felt this letter was about our son and the experience … No recommendation of change … Basically digging themselves out of a hole … “Rest assured, we have thoroughly investigated this.” I'm like, yeah, how? Doing what? Talking to who?

(Participant 7, consumer, interview)

I don't think people care so much about the bureaucratic process. What people care about is knowing that if you've made a complaint, you screwed yourself up and opened yourself to criticism and you've done it because whatever happened to you distressed you enough that you don't want it to happen to someone else.

(Participant 18, consumer, interview)

Participants noted that review reports sometimes had inaccuracies or missing details and often did not reflect a system‐focused approach, with findings blaming individuals. Ensuring reviews were reflective of a restorative just culture was important to participants.

…there was one [note] that was retrospectively written after my complaint. It had inaccuracies. It was misleading in some parts, and it had very integral information, at least from my perspective, missing. He [the clinician] said observations were fine … That's inaccurate … It's not true.

(Participant 2, consumer, interview)

Dismissing the consumer voice, minimising or mis‐conceptualising their experience, asking limited questions about the consumer experience, and minimising their involvement in the review process were noted as issues in the PSI review process. Participants explained how these issues required consumers to advocate for themselves to be involved in the review, which was not always well received by health services if they were perceived to be challenging the process. Participants noted that while consumers may receive open disclosure, they may not necessarily be involved in the review process or even be asked to provide input during the data collection phase of the review. In addition, several participants reported limited acknowledgement of the incident or response to consumer concerns by the hospital.

From the minute [the incident] happened, and we were told the investigation was actually going to happen, we weren't contacted by the hospital ever again.

(Participant 8, consumer, interview)

[the clinician] sent an e‐mail back to patient experience within less than a day from when I made the complaint, and then they had a meeting with me. So, there was no consultation with me about my perspective at all.

(Participant 2, consumer, interview)

Participants perceived the PSI review process to be slow and under‐resourced. They expressed concern about the quality of recommendations, as well as poor planning throughout the process. Participants noted dissatisfaction with the ineffective changes achieved from reviews and the limited resolution of their review.

…I did get a letter … I remember at the time getting it in the mail and I have no idea how long afterwards. It was a long time, it could have been close to 12 months after the event….

(Participant 7, consumer, interview)

Participants felt that the inclusion of an independent reviewer on the PSI review panel would enhance the rigour and their trust in the process, identifying that organisations investigating themselves posed issues of bias. A level of independence and regulation of the review process was seen to improve consumer confidence in the review process.

You want to know that the people sitting around the table doing the interview aren't just not involved but are from other health services as well. I think that that's so that there's a broad perspective on what's actually happened … It shouldn't be just done internally. It should be done from, you know, a couple of people, if possible, from an external organisation. They [the independent reviewer] don't come from … that health service … They actually bring a new perspective.

(Participant 13, consumer representative, interview)

Reimbursement for ad hoc expenses such as parking and transport was also suggested to minimise barriers to consumers engaging in the review process and make them feel more supported. At the system level, this support would ensure consumer engagement is properly resourced with financial and logistical support.

We haven't mentioned about just some of the practical supports around, say like cost of transport or parking or whatever sorts of … really practical things that can end up being an additional burden on the on top of … what is kind of a burdensome sort of process for people. That just that eases the load a bit.

(Participant 16, consumer representative, focus group)

In summary, during the review, consumer support focused on transparency and open communication about the process, progress and findings, while being attentive to consumers' emotional and financial burdens. Communication should be undertaken with trauma‐informed and culturally appropriate approaches, designed to safeguard against compounded harm. Review outputs should be provided in accessible language, particularly for people from marginalised groups, alongside tailored support, including counselling services. Other practical considerations included reimbursement for out‐of‐pocket expenses, such as transport and parking, to make consumer engagement more accessible and inclusive.

### 3.4 Consumer Needs After the Review

After the review had been completed and the report written, participants felt that consumers ought to be offered a meeting to discuss the report and findings, and to understand the actions taken to prevent the PSI from reoccurring. The meeting should be held at a venue acceptable to the consumer. Ensuring a meeting like this is documented was also seen as vital to ensure consumers are able to absorb and review the information provided after the meeting.

Sometimes, especially if you're a bit heightened, you don't necessarily absorb what is said to you in a face‐to‐face meeting, or you can't necessarily remember it clearly. So, I think that documentation is pretty important.

(Participant 5, consumer representative, focus group)

Participants deemed it fundamental that the consumer voice is listened to and their needs addressed, with this reflected in the report and recommendations. Participants felt it was important that they could provide feedback about the recommendations before the report was finalised. Involving consumers at this stage signals respect for their contribution, helps avoid misinterpretation of their experiences, and can reduce the risk of further harm or frustration.

[there is] Always that question of how will I know that you're [the health service] doing what you said you would do, like how would a consumer be able to have confidence that this thing [recommendation implementation] that was meant to happen, happened?

(Participant 5, consumer representative, focus group)

There is also a need for follow up reporting on what concrete changes were implemented as a result of the review. Doing so supports a restorative culture and reinforces trust in the system after harm has been experienced.

I would like to have an update like three months later in terms of what I've actually implemented and how I've implemented that. And I would like to understand how they would make sure this isn't happening to anybody else….

(Participant 17, consumer, interview)

I just think they [consumers] need to be updated on…how something can be prevented from happening again. If it's a change in legislation, if it's changing policy procedure. Just some practical workings of how, you know, it's not going to happen again.

(Participant 22, consumer representative, interview)

A couple of participants believe that enhanced follow‐up about the implementation of recommendations and associated outcomes would ensure greater accountability for the health service. This follow‐up process closed the feedback loop to make the process visible to participants, without which, the review process may be perceived as performative. It also indicates that patient safety reviews should include structured mechanisms for consumer involvement for tracking, reporting and communicating implementation progress and outcomes rather than ending at the point of report completion.

You want accountability, and I don't think that element is always there in the way that they do the investigations because they are too busy worrying about blaming people … and then comes the trust issue, then comes the whole, you know, they're not open and transparent, even though they say they are.

(Participant 14, consumer representative, interview)

After the review, consumers need to be kept informed about the findings, and any changes implemented with periodic updates provided, such as at 6 and 12 months after the review. Health organisations should have clear mechanisms to track, report and communicate implementation progress. Consumers should be offered opportunities to discuss these updates at times convenient for them to ensure ongoing transparency and accountability beyond the conclusion of the review.

## 4 Discussion

This study identified a multitude of consumer needs before, during, and after PSI reviews. Essentially, responses to PSIs, need to be more patient‐centred, through greater engagement with, and consideration of consumer needs. Our study conceptualises consumer needs in interrelated dimensions, including: emotional and psychological needs (including a need for empathy and compassion, and a just and restorative approach or process [8, 25]); practical needs (including access to clear and timely information, a dedicated contact and financial support covering parking and transport); institutional care and processes (related to structural mechanisms to report, track and provide feedback about the review process and outcomes of change); and intersectional needs, specifically foregrounding the needs of CALD communities and people with disabilities. These needs are applicable throughout all stages of the response process.

There is growing recognition in the literature that points to the importance of consumer involvement and needs in relation to reviews, for example, internationally [26, 27, 28], England [29, 30], Scotland [31], Netherlands [32], Norway [33], Sweden [34], New Zealand [35], Canada [36], and Australia [25]. Many of those studies [15] conceptualise consumers' needs related to a just and restorative culture [13, 26]. Even so, there is a gap in practice ‐ participants in our study continued to describe frustrations that their voices were not being heard, that they experienced powerlessness within the review process, and were even less engaged after the report was written. Partly, these issues are due to the pre‐existing structural barriers, for example, power dynamics within the health system [15, 37]. Our study and others [31, 38] show that disclosure is more than a procedure in place, but relational, where actively listening to consumers' needs with empathy and compassion should be demonstrated. Honesty, acknowledgement, and clear communication of what to expect are essential [25, 38]. Additionally, consumers' needs and voices, therefore, should be welcomed and heard earlier. This patient‐centred approach could increase the consumers' perception of trust in the healthcare system [12].

Much of the existing literature focuses on the disclosure process or the review [38, 39, 40], but less so on the whole response process. To the best of the authors' knowledge, this is the first paper to articulate consumers' needs for temporally constructed, interconnected experiences, emphasising that the completion of the report should not be considered closure. It resonates with a Dutch study [32] that PSI reviews should be considered as part of the existing relationship between consumers and the health system, and part of ongoing care.

Similar to other research, our study suggests that the response to PSI reviews may cause compounded harm if not addressed appropriately [2, 41], and that consumers' experience of harm is cumulative and can unfold over time through waiting, silence or poorly handled responses rather than the PSI alone. Right after the PSI, consumers often feel vulnerable and overwhelmed after a PSI, not knowing which steps they should take or where they can get support [8]. At the same time, consumers may feel initially hopeful that their voices will be heard during the review and may help to make a positive change [8]. Consumer needs should be recorded and documented, which is different from open disclosure. Poor communication, a lack of support from the organisation, feeling like their attempts to escalate reports of a PSI are unheard, and delays to PSI review, however, can taint this optimism [8, 41]. The heavy emotional and financial labours of navigating the layers of communication are evident in this study.

Consumers in this study, and elsewhere [31], want to feel they are being listened to and included in the review process, through a consumer‐centred focus, and they should have an opportunity to detail and document their experiences in a personalised way. Previous research has identified that being listened to can help consumers to regain trust in the healthcare system and enhance their satisfaction with the review process [31]. Part of being listened to includes being asked about their preferences and needs throughout the review and feeling that they could improve their learning by providing their unique insights [31]. Communication and support, therefore, should be offered at different points during the process, with the option to defer, pause or re‐engage as needed. Assistance from DFCs or liaison officers, similar to the processes established by the Clinical Excellence Commission in NSW, Australia [42] (see Box 1), would support consumers' needs and rights, with timely communication. There is, however, a tension between the constrained timelines of reviews and the flexibility to understand consumers' needs, levels of involvement and preference of engagement (including timing, formats or communication channels) to do so.

Box 1 **Dedicated family contact (DFC) for patient safety incident reviews in New South Wales, Australia [42].**

A DFC is a primary staff contact who:

- Provides continuity during a serious adverse event review (SAER) and beyond if required;
- Establishes rapport, credibility and trust with consumers;
- Understands the consumers' preferred communication approach and concerns;
- Provides practical assistance, for example, a social worker, an interpreter and parking;
- Liaises with the Serious Adverse Event Review and Open Disclosure team;
- Explains incident management processes and timelines;
- Recognises/supports cultural needs, for example, Aboriginal, culturally and linguistically diverse (CALD) communities.

Additionally, tailored support is needed for CALD consumers. Intersectional factors related to socioeconomic status, culture, language and disability [43] add extra layers of complexity in how consumers experience institutional care and articulate their needs [44, 45, 46]. Consumer involvement, therefore, is not only relational but also cultural [47, 48]. Equity and inclusion should also be considered across the whole response process.

Our findings show that, as well as some consumers experiencing a lack of compassion and empathy during the review process, there may be limited acknowledgement of and accountability for the PSI. This is critical, as participants identified that a key driver of consumer involvement in a review is the aim to reduce the occurrence of further PSIs. Although consumers may seek answers from review reports, they are often unsatisfactory, seeming defensive, or lacking accountability, neglecting opportunities to learn from the PSI, with seemingly arbitrary recommendations resulting from a review, a finding supported by the literature [8, 32, 49]. Transparency and accountability are not administrative details but a necessity to safeguard against compounded harm during the review and to reduce reoccurrence of PSIs [2, 50].

In addition to system‐focused review approaches [13, 18] that emphasise technical and organisational processes, a patient‐centred relational approach equally prioritises consumers' need for fairness, transparency and accountability. These two orientations should not be understood as contradictory, as both share a commitment to learning and improving from harm and preventing further harm [31]. Accountability can also be enhanced by incorporating some independence within the review, for example, through the inclusion of CRs [15, 17] and external reviewers on review teams [13].

Our study also demonstrates that consumers need reports written in language that is suitable for consumers [18]. This should be treated as a consumer right, rather than something that can lost in bureaucratic processes. Consumers' voices should be valued, reflected in the report, and taken into account when developing aligned recommendations. These processes must also be accessible to people from CALD communities or with disabilities.

Immediately following the review, consumers should be provided with opportunities to discuss the report and findings, which should align with their preferences (e.g., venues, formats and communication channels). This process should ensure consistency in reflecting consumer needs, clearly documenting them in the report, and opportunity to provide feedback on the recommendations before the report is finalised. Involving consumers at this stage signals respect for their contribution and helps avoid misinterpretation of their experiences. Such processes can also reduce the risk of further harm or frustrations [50].

Participants in our study asked for a rethinking of how patient safety reviews are closed, emphasising their desire to be involved in the learning process and to see changes made as a result of their lived experiences. This finding is missing in the PSI review literature. This call aligns with other research advocating for partnering with consumers throughout the review [51] and views this as part of the ongoing relationships with health organisations. Follow‐up reporting on what concrete changes were implemented as a result of the review supports a restorative culture and reinforces trust in the system after harm has been experienced. The consumer's continued involvement, therefore, increases the organisation's accountability to implement recommendations. It would require a structured mechanism for consumers to be involved in the tracking, reporting and communicating of changes to be implemented [2, 50].

To address these challenges, the findings from this study, in conjunction with other arms of this project [13, 15, 16, 17, 18], will inform the development of Best Practice Principles to enhance learning and improvement from PSIs, better support consumers, and ultimately contribute to reducing patient safety risks. Proposed improvements are outlined in Table 2.

**Table 2:** Best Practice Principles for supporting consumers' needs associated with a patient safety incident (PSI) review.

Organisations explicitly ask what consumers need from the PSI Review, including what questions they wish to be answered about the PSI, what outcomes matter most to them, and how and whether they would like to be involved at different stages of the process. PSI reviews are conducted using trauma‐informed and culturally safe approaches towards engagement with consumers across all stages, including flexibility in timing, mode of engagement and communication, sensitive to emotional readiness, cultural practices, disability related needs and family circumstances. PSI reviews consider the perspectives of consumers throughout the review and acknowledge power dynamics to ensure their perspectives are taken seriously, including recording their perspective and experience of the PSI, and documenting and incorporating their feedback within the recommendations to prevent future harm. PSI reviews have clear and systematic methods of transparently reporting the progress of the review to consumers, including a clear outline of the plan and timeline of the review, and the methodology used, to ensure they are fully informed and regularly updated on review progress. PSI reviews produce transparent and comprehensive reports in language appropriate to consumers, with clear explanations of medical jargon throughout, that clearly outline the findings and recommendations proposed, as well as the progress in implementing recommendations and outcomes. Duty of Candour/Open Disclosure is conducted for serious PSIs, including access to PSI Review reports, and opportunities to discuss the report and ask any questions. Communication with consumers is empathetic and personalised. Organisations provide enhanced support for consumers through the consistent use of a patient liaison officer (or Dedicated Family Contact). Support may also include practical measures such as remuneration for psychological support, counselling and parking. Health organisations should have structured mechanisms for consumers' involvement for tracking, reporting and communicating implementation progress and outcomes rather than ending at the point of report completion.

### 4.1 Strengths and Limitations

This study incorporated the experiences of a range of consumers and CRs across four states and territories. The triangulation of perspectives from different health services added to the rigour of the research, as did the use of multiple coders to analyse the data. This study has several limitations. Participants were recruited using a non‐probability sampling strategy, combining purposive and convenience sampling with self‐selection. We acknowledge that this strategy may have introduced bias, in particular favouring individuals who are already engaged in the health system and its consumer networks. Some recruitment was conducted through CR networks, which disseminated the study invitation on behalf of the research team. As a result, we did not purposively sample for a specific population, nor did we collect demographic information related to background, such as gender, sexuality, disability status, age, or cultural or linguistic background. We therefore were unable to determine the extent to which our findings reflect the experiences of these populations.

## 5 Conclusion

This study has highlighted the needs of consumers before, during and after the PSI review. Ensuring the response to PSIs is more patient‐centred, through more meaningful engagement and consideration of consumers' emotional, psychological, communication and transparency, accountability and practical needs, is an important change. A patient‐centred relational approach, complementing systems‐based reviews, will contribute to improving learning and improvement following a PSI, enhance the consumer experience, including reducing compounded harm, and support them to regain trust in the healthcare system.

## Author Contributions

**Mia Bierbaum:** writing – original draft, writing – review and editing, validation, investigation, formal analysis, methodology. **Yinghua Yu:** writing – review and editing, investigation, formal analysis, methodology, validation. **Charlotte J. Molloy:** writing – review and editing, project administration, methodology, validation. **Lorelle Bowditch:** data interpretation, validation, writing – review and editing. **Peter D. Hibbert:** conceptualisation, investigation, funding acquisition, methodology, writing – review and editing. All authors discussed the results and contributed, reviewed, and revised the final manuscript.

## Ethics Statement

The study was conducted in accordance with the Northern Sydney Local Health District (NSLHD) Human Research Ethics Committee (approval number: 2023/ETH02341).

## Consent

All patients were informed that participation was voluntary and provided written informed consent before the interview.

## Conflicts of Interest

The authors declare no conflicts of interest.

## Supporting information

Supporting File 1.

Supporting File 2.

## Data Availability

All the data is confidential due to ethical considerations.
