# Supplementary material for: What Do Consumers Need Before, During and After a Patient Safety Incident Review? A Qualitative Study
Source: Health Expect. 2026 Mar 26;29(2):e70646. doi: 10.1111/hex.70646 (PMC13125722; doi:10.1111/hex.70646)
Supplement: Supplementary file 1 — Supporting File 1. [file HEX-29-e70646-s002.docx]

**Supplementary file 1**

**Consolidated criteria for reporting qualitative studies (COREQ): 32-item checklist**

**Source:** *Tong, Allison, Peter Sainsbury, and Jonathan Craig. "Consolidated criteria for reporting qualitative research (COREQ): a 32-item checklist for interviews and focus groups." International journal for quality in health care 19.6 (2007): 349-357.*

| **No** | **Item** | **Guide questions/description** | **Page** |
| --- | --- | --- | --- |
| **Domain 1: Research team and reflexivity** |  |  |  |
| **Personal Characteristics** |  |  |  |
| **1.** | **Interviewer/facilitator** | **Which author/s conducted the interview or focus group?** | **5** |
| **2.** | **Credentials** | **What were the researcher's credentials? *E.g. PhD, MD*** | **5** |
| **3.** | **Occupation** | **What was their occupation at the time of the study?** | **5** |
| **4.** | **Gender** | **Was the researcher male or female?** | **5** |
| **5.** | **Experience and training** | **What experience or training did the researcher have?** | **5** |
| **Relationship with participants** |  |  |  |
| **6.** | **Relationship established** | **Was a relationship established prior to study commencement?** | **5** |
| **7.** | **Participant knowledge of the interviewer** | **What did the participants know about the researcher? e*.g. personal goals, reasons for doing the research*** | **5** |
| **8.** | **Interviewer characteristics** | **What characteristics were reported about the interviewer/facilitator? e.g. *Bias, assumptions, reasons and interests in the research topic*** | **5** |
| **Domain 2: study design** |  |  |  |
| **Theoretical framework** |  |  |  |
| **9.** | **Methodological orientation and Theory** | **What methodological orientation was stated to underpin the study? *e.g. grounded theory, discourse analysis, ethnography, phenomenology, content analysis*** | **5** |
| **Participant selection** |  |  |  |
| **10.** | **Sampling** | **How were participants selected? *e.g. purposive, convenience, consecutive, snowball*** | **4** |
| **11.** | **Method of approach** | **How were participants approached? e*.g. face-to-face, telephone, mail, email*** | **4** |
| **12.** | **Sample size** | **How many participants were in the study?** | **6** |
| **13.** | **Non-participation** | **How many people refused to participate or dropped out? Reasons?** | **6** |
| **Setting** |  |  |  |
| **14.** | **Setting of data collection** | **Where was the data collected? e*.g. home, clinic, workplace*** | **5** |
| **15.** | **Presence of non-participants** | **Was anyone else present besides the participants and researchers?** | **4** |
| **16.** | **Description of sample** | **What are the important characteristics of the sample? *e.g. demographic data, date*** | **6** |
| **Data collection** |  |  |  |
| **17.** | **Interview guide** | **Were questions, prompts, guides provided by the authors? Was it pilot tested?** | **5** |
| **18.** | **Repeat interviews** | **Were repeat interviews carried out? If yes, how many?** | **NA** |
| **19.** | **Audio/visual recording** | **Did the research use audio or visual recording to collect the data?** | **5** |
| **20.** | **Field notes** | **Were field notes made during and/or after the interview or focus group?** | **5** |
| **21.** | **Duration** | **What was the duration of the interviews or focus group?** | **6** |
| **22.** | **Data saturation** | **Was data saturation discussed?** | **5/6** |
| **23.** | **Transcripts returned** | **Were transcripts returned to participants for comment and/or correction?** | **NA** |
| **Domain 3: analysis and findingsz** |  |  |  |
| **Data analysis** |  |  |  |
| **24.** | **Number of data coders** | **How many data coders coded the data?** | **5** |
| **25.** | **Description of the coding tree** | **Did authors provide a description of the coding tree?** | **5** |
| **26.** | **Derivation of themes** | **Were themes identified in advance or derived from the data?** | **5** |
| **27.** | **Software** | **What software, if applicable, was used to manage the data?** | **5** |
| **28.** | **Participant checking** | **Did participants provide feedback on the findings?** | **NA** |
| **Reporting** |  |  |  |
| **29.** | **Quotations presented** | **Were participant quotations presented to illustrate the themes / findings? Was each quotation identified? e*.g. participant number*** | **6-15** |
| **30.** | **Data and findings consistent** | **Was there consistency between the data presented and the findings?** | **6-15** |
| **31.** | **Clarity of major themes** | **Were major themes clearly presented in the findings?** | **6-15** |
| **32.** | **Clarity of minor themes** | **Is there a description of diverse cases or discussion of minor themes?** | **6-15** |
