# Supplementary material for: What Do Consumers Need Before, During and After a Patient Safety Incident Review? A Qualitative Study
Source: Health Expect. 2026 Mar 26;29(2):e70646. doi: 10.1111/hex.70646 (PMC13125722; doi:10.1111/hex.70646)
Supplement: Supplementary file 2 — Supporting File 2. [file HEX-29-e70646-s001.docx]

Stage 4 Interview Schedule Consumers

**Research Question**: How should we decide what to investigate, and to what level, to maximise learning to improve patient safety – making best use of the limited resources available?

**End Goal:** to inform the development of Triage Tool and Guidance- evidence based, feasibility tested, tools and guidance to determine what to investigate to what level for maximum value

**Interview / Focus Group Goal:** To canvas view from a consumer about what they need from the overall investigative process, and how those needs could be met.

| **Interview/ Focus Group Context:**   - Introduce the project and research questions - Introduce Restorative Just Culture - Goal is to create more value and learning from investigative process for all stakeholders including Consumers | **Participants:**   - Consumers |
| --- | --- |
|  | |
| **Interview/ Focus Group Questions** | **Purpose** |
| 1. When is an investigation required/ not required from a consumer’s perspective- What criteria should determine this? | Identify what could we stop doing while still meeting needs of consumers as well as the criteria for determining |
| 1. When is an investigation needed – what features or processes should be integrated into the process to ensure consumer needs are met? | Identify features of the investigation process necessary for meeting consumer needs |
| 1. What do consumers need from the investigative process - Is the current process meeting this? | Establish Baseline – what’s working well/not so well |
| 1. How could the needs of Consumers be met – outside of a formal investigation process? 2. What other mechanisms could fulfill this purpose? (E.g., Restorative Just Culture (RJC) process) | Establish if some needs could be met in another way – i.e., not through full investigation – that might be a better use of resources or better meet consumer need. |
| 1. How would a RJC approach be received by consumers? (interview for consumer perspective of events; and lessons consumers feel need to be learned; then gather any questions that would like answered within the review process. Follow up: Meet to share findings or review, structured interaction, answers to questions raised, feedback on actions taken. Consumer provides post incident feedback) | Understand the appetite for RJC from consumers and specifically how this might address consumer needs when compared to traditional approaches.  Understand how this would complement or replace parts of a formal investigation for consumers. |
| 1. After the investigation has been completed, the health service needs to implement the recommendations. What are the needs of consumers during this time? | Identify features of implementing recommendations process necessary for meeting consumer needs. |

| **Research Sources:**   - Behavioural insights into patient motivation to make a claim for clinical negligence Final report by the Behavioural Insights Team August 2018- NHS Resolution (NHS) - Hibbert, P., Thomas, M.J.W., Deakin, A., Runciman, W., and Braithwaite, J. (2016) Final Report: Sentinel Event Research Project. Australian Patient Safety Foundation. A report submitted to the Victorian Department of Health and Human Services, Melbourne, Victoria, Australia (VIC) - Vincent C, Carthey J, Macrae C, Amalberti R. Safety analysis over time: seven major changes to adverse event investigation. Implementation Science: IS. 2017 Dec;12(1):151. DOI: 10.1186/s13012-017-0695-4. PMID: 29282080; PMCID: PMC5745912 (V&A) - Turner K, Stapelberg NJ, Sveticic J, Dekker SW. Inconvenient truths in suicide prevention: Why a Restorative Just Culture should be implemented alongside a Zero Suicide Framework. Aust N Z J Psychiatry. 2020 Jun;54(6):571-581. doi: 10.1177/0004867420918659. Epub 2020 May 8. PMID: 32383403. (TSSD) |
| --- |
